# Insecticidal Effects of Fumigants (EF, MB, and PH_3_) towards Phosphine-Susceptible and -Resistant *Sitophilus oryzae* (Coleoptera: Curculionidae)

**DOI:** 10.3390/insects10100327

**Published:** 2019-09-30

**Authors:** BongSu Kim, Ja-Eun Song, Jeong Sun Park, YoungJu Park, Eun-Mi Shin, JeongOh Yang

**Affiliations:** Department of Plant Quarantine, Animal and Plant Quarantine Agency (APQA), Gimcheon 39660, Korea; bskim79@korea.kr (B.K.); sje626@gmail.com (J.-E.S.); jungsun5009@naver.com (J.S.P.); bangjune@naver.com (Y.P.); eunmishin@korea.kr (E.-M.S.)

**Keywords:** phosphine, resistance, *Sitophilus oryzae*, ethyl formate, methyl bromide

## Abstract

This study was conducted to evaluate the insecticidal effects of ethyl formate (EF), methyl bromide (MB), and phosphine (PH_3_) fumigants against PH_3_-susceptible and -resistant strains of the rice weevil (*Sitophilus oryzae*), a major rice pest. The highest lethal concentration time 50 (LCt_50_) values of the PH_3_-susceptible strains were 255.797, 21.104, and 6.171 mg h L^−1^ for EF, MB, and PH_3_, respectively, at pupal stage. The highest LCt_50_ values of the PH_3_-resistant strains were 149.028 and 48.170 mg h L^−1^ for EF and PH_3_, respectively, at late larval stage, and 43.520 mg h L^−1^ for MB at pupal stage. In comparison to the susceptible strains, the PH_3_-resistant strain collected in South Korea had a resistance level 4 to 56 times higher. Use of the major quarantine fumigants EF, MB, and PH_3_ indicated the existence of PH_3_-resistant rice weevils in South Korea for the first time.

## 1. Introduction

Stored pests are known to cause damage to stored products, such as reduced grain weight, quality, commercial value, and seed survival [1]. More than 600 species of insects infesting stored grains belong to Coleoptera, among which the genus Sitophilus includes several important grain pest species [2,3,4,5]. *Sitophilus oryzae* (L.) (Coleoptera: Curculionidae) reduces the amount and quality of stored grains and causes economic losses [1,6]. *S. oryzae* females make holes and lay an egg, and larval and pupal period is spent hidden in the stored grain [6]. *S. oryzae* larvae feed on proteins and vitamins by consuming the germ of cereals, and adults largely reduce the carbohydrate content by consuming the endosperm of the grains [1,7].

Methyl bromide (MB) and phosphine (PH_3_) fumigants are mainly used for fumigation of grain at storages and silos to control pests damaging stored grains [8]. MB is an old fumigant discovered in 1932 that has been used in virtually every field of disinfection and disinfestation, such as fruit, grains, wood, and necessities [9]. However, it has been designated as an environmentally destructive substance causing ozone depletion [10], and its use has been completely prohibited except for quarantine and pre-shipment purpose under the Montreal Protocol [11,12]. PH_3_ is known to spread rapidly during fumigation, has low fumigant residue after treatment, and has no side effects on seed survival [13,14]. However, PH_3_ resistance has been found in India, China, Vietnam, Morocco, Brazil, and Australia. In each country, *S. oryzae* has been reported to be phosphine-resistant [15,16,17,18,19,20,21].

As an alternative to these fumigants, the efficacy of ethyl formate (EF) against stored product insects have been investigated in a wide range of studies [22,23,24]. EF is a low-molecular-weight volatile material characterized by little residue [25] and occurs naturally in a variety of products such as beer, rice, and beef [26]. It has been used for dry fruit insect control since 1927 and has also been used for the disinfection of cereals, stored dry beans, tomatoes, and preserved foods [27,28]. In the United States, pesticides have been registered [29] for the control of pests in storage products such as *Tribolium confusum* and *Ephestia figuliella* in raisins [30].

*S. oryzae* is an important pest in the management of postharvest agricultural products. Phosphine resistance of *S. oryzae* has risen sharply to 75% in developing countries by 2000 [20], requiring the management of resistance. Therefore, this study was conducted to investigate the resistance levels of the newly discovered PH_3_-resistant rice weevils of South Korea and compare these resistance levels to those of susceptible strains. EF, MB, and PH_3_ fumigants were used to provide baseline data on rice weevil control through a comparison of treatments currently recommended by quarantine.

## 2. Materials and Methods

### 2.1. Insects

**Figure 1:**
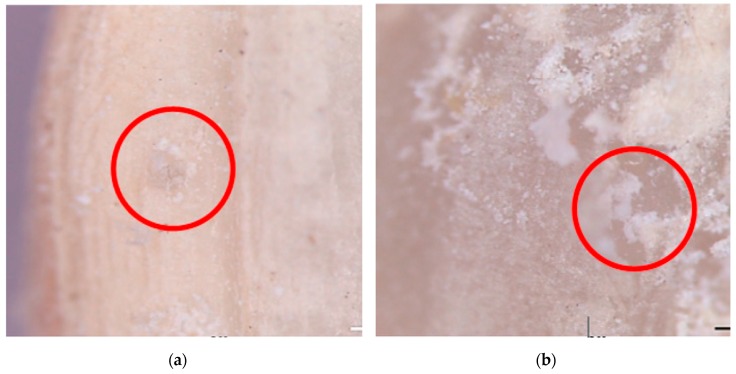

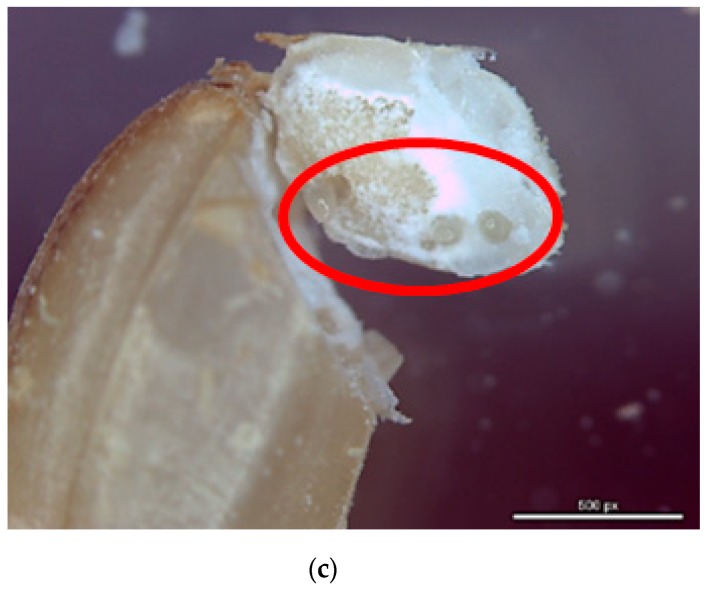
Identification method of rice weevil infected grain. (**a**) Egg of *Sitophilus oryzae*; (**b**) an egg covered with rice; (**c**) inside egg with removed surface of rice.

PH_3_-susceptible strain (control strains) of *S. oryzae* was reared at Murdoch University (WA, Australia), and the PH_3_-resistant strain of *S. oryzae* was reared at Chungbuk National University (Chungcheongbuk-do, South Korea). All tested insects were brought to the Plant Quarantine Technology Center before experiment (Gyeongsangbuk-do, South Korea). The breeding conditions were maintained at a temperature of 27 ± 1 °C and relative humidity of 50–60% in a brown rice feed at the Plant Quarantine Technology Center. The presence or absence of *S. oryzae* eggs were confirmed by thinning the area covered with gelatinous fluid near the rice grain (Figure 1). *S. oryzae* eggs were used in the experiment within 2–3 days after oviposition of *S. oryzae* adults. The larvae within 1–2 and 22–25 days after hatching were used as early and late larvae, respectively. Pupae were collected within 3–4 days after pupation. Adult of *S. oryzae* were grown for 10–20 days after emergence. The inside of the rice was checked to determine whether larvae or pupae were present. It was difficult to observe the inside of the brown rice, so a stereomicroscope (MDG33, Leica, Wetzlar, Germany) was used. All insects were inoculated on an insect breeding dish (Φ 5.5 cm × 1.5 cm, SPL, Pocheon, Gyeonggi-do, Korea) and fed 2 g of brown rice.

### 2.2. Fumigants

PH_3_ was purchased as ECO2Fume (PH_3_ 2% + CO_2_ 98%) from Cytec (Sydney, NSW, Australia), and EF was purchased as liquid EF (EF 97%) from Aldrich Chemical Company Inc. (St. Louis, MO, USA). MB was used as a control, and Youngil MB (MB 98.5%) of Nonghyup Chemical (Seongnam, Gyeonggi-do, Korea) was used.

### 2.3. Fumigation System

Fumigant treatment was performed at the Plant Quarantine Technology Center. The fumigant treatment was applied in a fumigation chamber at a temperature of 20 ± 1 °C and a relative humidity of 60 ± 10%, and fumigation was carried out in a 12 L glass desiccator (DWK Life Sciences, Mainz, Germany). Tested insects were put in 100 × 40 mm size insect breeding dish (SPL, Pocheon, Gyeonggi-do, Korea) before fumigation. More than 30 insects were carried in each dish and three dishes were used for each test as replicates.

PH_3_-susceptible *S. oryzae* was treated at a concentration of 0.01 to 4.00 mg/L for 20 h, but in the case of PH_3_-resistant *S. oryzae*, a high concentration exceeding 4 mg/L is required, so concentration was fixed to 0.50 mg/L and the treatment time was varied from 24 h to 504 h. EF was applied at 10 to 150 mg/L for 4 h, and MB was applied at 3 to 25 mg/L for 4 h. Test of PH_3_ resistance was performed according to the Food and Agriculture Organization (FAO) criteria [31]. According to FAO test, a population was judged to have resistance when more than 2 individuals survived for 20 h at PH_3_ 0.04 mg/L. EF and MB treatments were observed at 24 h after fumigation, and PH_3_ treatment was observed at 72 h after fumigation due to the knock-down phenomenon. The insecticidal rate was determined by touching an insect body with a microscopic needle. It was judged as dead if there was no movement using a microscope (MDG33, Leica, Wetzlar, Germany).

### 2.4. Measurement of Fumigant Concentrations

To investigate the actual concentration in the treated samples, 60 mL of sample gas was extracted from all experimental groups using a 60 mL syringe and 1 L Tedlar bag (SKC, Dorset, United Kingdom). EF and MB samples were taken at 30 min and 1, 2, and 4 h, and PH_3_ samples were taken at 30 min and 1, 4, and 20 h after fumigation. If fumigation time was longer than 20 h, samples were extracted at 24-h intervals until the completion of the experiment.

The concentrations of EF and MB were measured using an Agilent GC 7890A equipped with a flame ionization detector (FID) after separation on an Rtx-5 column (15 m × 250 μm × 1 μm, RESTEK, Bellefonte, PA, USA) operating in split mode (10:1). The PH_3_ concentration was determined using an Agilent GC 7890A equipped with a flame photometric detector (FPD) and HP-PLOT/Q (30 m × 530 μm × 40 μm, Agilent, Santa Clara, CA, USA) operating in split mode (10:1). The injector and oven temperature were 200 °C. The detector temperature was 250 °C. The injection volumes and flow rates of EF, MB, and PH_3_ were 60, 60, and 20 μL and 1.5, 1.5, and 5 mL/min, respectively. The concentrations of EF, MB, and PH_3_ were calculated based on peak areas against external standards.

### 2.5. Determination of the Concentration × Time (Ct) of the Fumigants

Concentration is an important factor to determine efficacy of chemical insecticide, including fumigants. However, fumigation time also is an important factor particularly for the fumigant, although, importance can be varied by the kind of fumigant [32]. To examine the effect of both concentration and time, exposure of the fumigant was expressed as concentration × time (Ct) product. The Ct of fumigants was calculated using the equation of Monro [33].

### 2.6. Statistical Analysis

All experiments were performed with 3 replicates. Percent mortality and standard error (SE) were determined via an Excel v. 2007. The lethal concentration time 50 (LCt_50_) and 99 (LCt_99_) values of *S. oryzae* were obtained using the Probit analysis [34] program (CSIRO 1998), with logarithmic transformation of data.

## 3. Results

### 3.1. Fumigation of Phosphine-Susceptible S. oryzae

**Figure 2:**
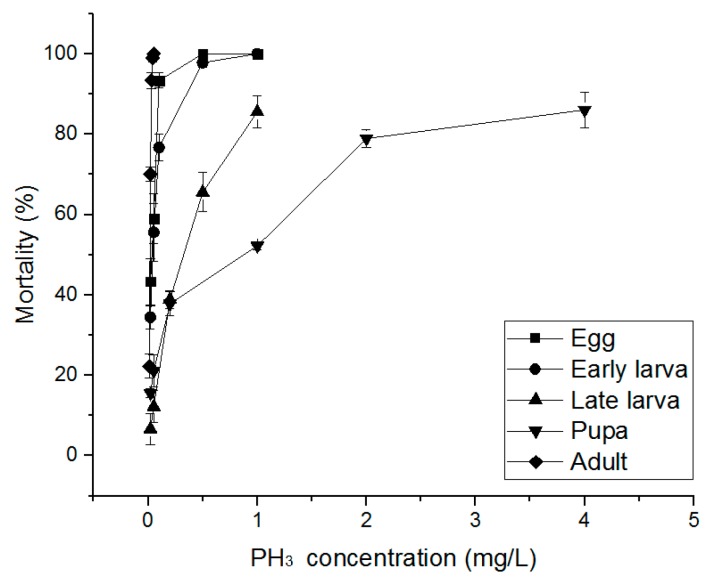
Mortality of phosphine susceptible *S. oryzae* exposed to phosphine (PH_3_) fumigant for 20 h at 20 °C in 12 L desiccator.

**Figure 3:**
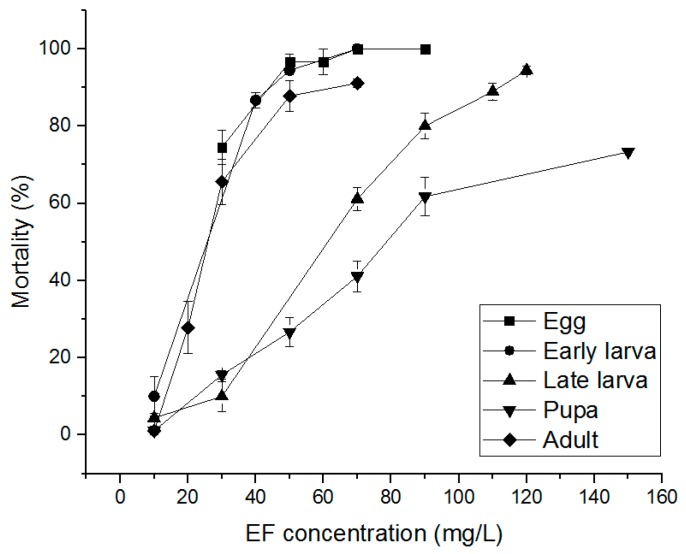
Mortality of phosphine susceptible *S. oryzae* exposed to ethyl formate (EF) fumigant for 4 h at 20 °C in 12 L desiccator.

**Figure 4:**
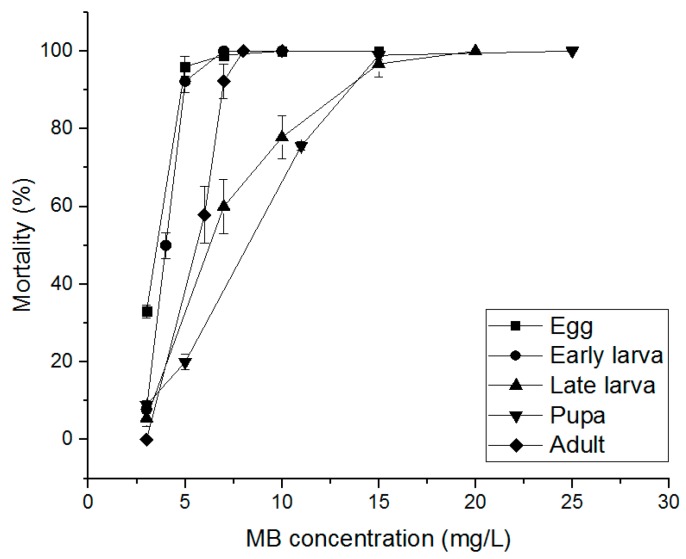
Mortality of phosphine susceptible *S. oryzae* exposed to methyl bromide (MB) fumigant for 4 h at 20 °C in 12 L desiccator.

The PH_3_ susceptibility-related effects of PH_3_, EF, and MB fumigants on *S. oryzae* eggs, early larvae, late larvae, pupae, and adults were evaluated. PH_3_ showed a 100% insecticidal rate against eggs, early larvae, and adults at 1.00 mg/L and an 85.56% insecticidal rate in late larvae at 1.00 mg/L (Figure 2). Pupae treated with 4.00 mg/L, the highest treatment concentration, showed an insecticidal rate of 86.04%. As a result of treatment with EF, eggs, early larvae, and adults showed insecticidal rates of 90% or more at an EF concentration of 70 mg/L (Figure 3). However, in the case of late larvae treated with 120 mg/L, the insecticidal rate was 94.43%. In the pupae, the highest treatment concentration of 150 mg/L was applied, but the insecticidal rate was as low as 73.30%. MB showed a 100% insecticidal rate at 10 mg/L for eggs, 8 mg/L for adults, and 7 mg/L for early larvae but 25 mg/L for pupae and 20 mg/L for late larvae (Figure 4). These results indicate that PH_3_-susceptible *S. oryzae* is most tolerant in the pupal and late larval stages. Among PH_3_, EF, and MB fumigants, LCt_50_ was the lowest (0.295 mg h/L) when treated with PH_3_, and the highest LCt_50_ (255.797 mg h/L) was obtained by treating the pupae with EF. PH_3_ showed a high insecticidal rate at a low concentration but had to be treated for a long time, and EF had to be treated at a high concentration, though the treatment time was short.

### 3.2. Evaluation of Phosphine-RESISTANT S. oryzae by Fumigation Time of Phosphine

**Figure 5:**
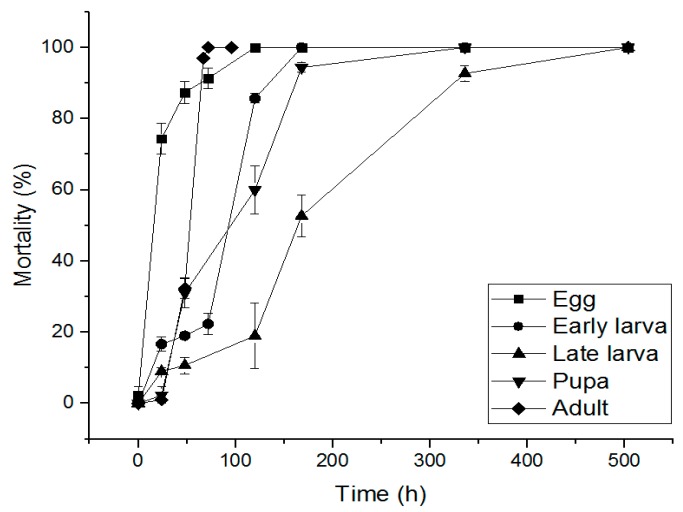
Mortality of phosphine resistant *S. oryzae* exposed to PH_3_ fumigant for 0.5 mg/L at 20 °C in 12 L desiccator.

Assessment of PH_3_ resistance was conducted according to the FAO criteria. *S. oryzae* collected from South Korea was treated with 0.04 mg/L PH_3_ for 20 h and compared with PH_3_-susceptible *S. oryzae* distributed from Australia. The PH_3_-susceptible *S. oryzae* showed a 98.3% insecticidal rate, and *S. oryzae* in South Korea was 21.0%. According to the FAO resistance evaluation criteria, *S. oryzae* in South Korea is considered to have resistance. The effect of PH_3_ on PH_3_-resistant *S. oryzae* was evaluated by setting the concentration to 0.5 mg/L and then by treatment time. As a result, the eggs, early larvae, late larvae, pupae, and adults showed 100% insecticidal rates when treated for 72, 120, 168, 336, and 504 h, respectively (Figure 5).

### 3.3. Alternative Fumigants for PH_3_-Resistant S. oryzae

**Figure 6:**
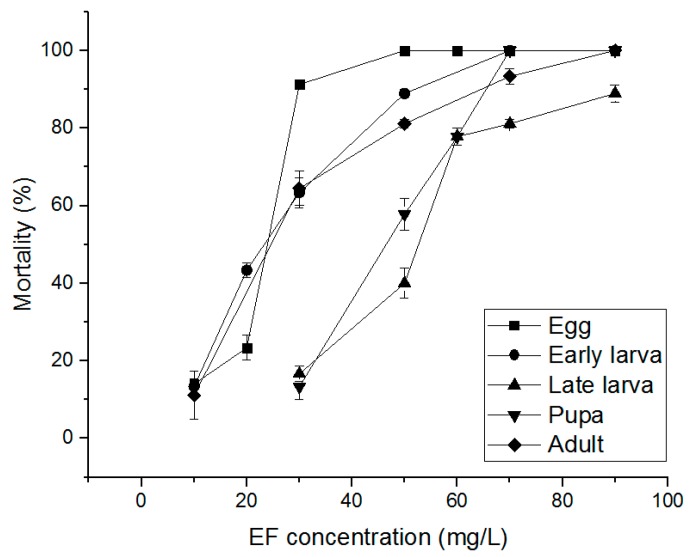
Mortality of phosphine resistant *S. oryzae* exposed to EF fumigant for 4 h at 20 °C in 12 L desiccator.

The effect of EF and MB fumigants on PH_3_-resistant *S. oryzae* eggs, early larvae, late larvae, pupae, and adults was evaluated. EF showed insecticidal rates of 100% at 70 mg/L for egg, early larvae, and pupae, 100% at 90 mg/L for adults, and 88.90% at 90 mg/L for late larvae (Figure 6).

**Figure 7:**
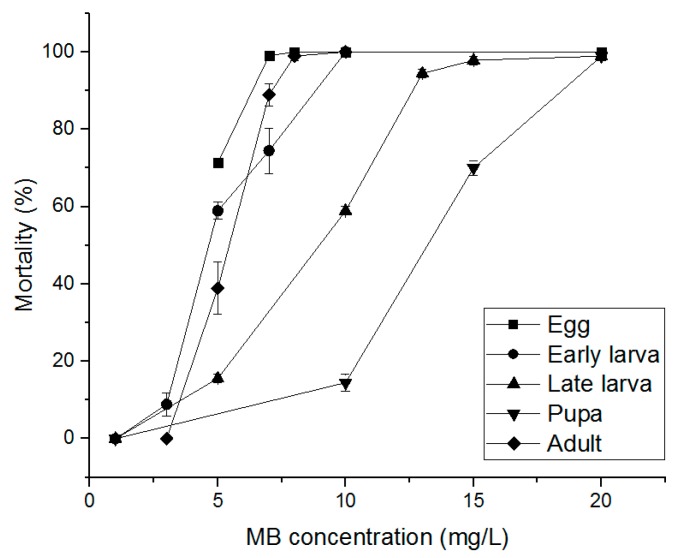
Mortality of phosphine resistant *S. oryzae* exposed to MB fumigant for 4 h at 20 °C in 12 L desiccator.

MB treatment showed a 100% insecticidal rate at 8 mg/L for eggs, 10 mg/L for early larvae and adults, whereas late larvae and pupae showed a 98.9% insecticidal rate at 20 mg/L (Figure 7). The LCt values were analyzed based on the effect of the fumigants on PH_3_-resistant *S. oryzae*. The LCt_50_ value of EF was the highest (149.028 mg h/L), and the LCt_50_ of MB was the lowest (25.840 mg h/L). The LCt50 of EF was 5.76 times higher than that of MB.

### 3.4. LCt Analysis of the PH_3_-Susceptible and PH_3_-Resistant Rice Weevils

The LCt_50_ and LCt_99_ values of the PH_3_-susceptible and PH_3_-resistant strains of rice weevils were analyzed based on the results of the effects of the three fumigants.

The LCt analysis results for EF showed that the LCt_50_ values of EF were confirmed to be the highest at pupal stage (255.797 mg h/L) and the lowest at early larval stage (60.110 mg h/L). The LCt_50_ values of EF on the PH_3_-resistant strains showed a different aspect with the previous one, that egg stage was the lowest (60.034 mg h/L) and late larval stage was the highest (149.028 mg h/L). PH_3_-resistant strain showed higher LCt_50_ than PH_3_-susceptible strain at early larval stages, but lower LCt_50_ at late larval and pupal stages, which are tolerant stages (Table 1).

The LCt_50_ values of MB for the PH_3_-susceptible strain were the highest at pupal stage (21.104 mg h/L) and the lowest at egg stage (9.997 mg h/L), and the LCt_50_ value of MB for the PH_3_-resistant strain were the highest at pupal stage (43.520 mg h/L) and the lowest at early larval stage (14.900 mg h/L). PH_3_-resistant strain showed higher LCt_99_ than PH_3_-susceptible strain at all stages except adult, but did not exceed 2.06-fold (Table 2).

As a result of LCt analysis for PH_3_, the LCt_50_ values of the PH_3_-susceptible strain were the highest at pupal stage (6.171 mg h/L) and the lowest at adult stage (0.295 mg h/L). The LCt_50_ values of the PH_3_-resistant strain were the highest at late larval stage (48.170 mg h/L) and the lowest at egg stage (6.595 mg h/L). The highest concentration needed to control the late larvae was different from that needed to control the susceptible strain. The concentration needed to control the eggs was 57.206 mg h/L, the concentration needed to control the early larvae was 107.914 mg h/L, the concentration need to control the late larvae was 241.311 mg h/L, the concentration need to control the pupae was 91.760 mg h/L, and the concentration needed to control the adults was 25.938 mg h/L. Control of rice weevil late larvae and pupae using PH_3_ should be considered (Table 3).

## 4. Discussion

In most species, egg and pupal stages are reported to be the most tolerant developmental stages to the fumigants, whereas *S. oryzae* pupae were the most tolerant [35]. In *Phthorimaea operculella*, pupal resistance was the strongest under EF treatment [36]. *Rhyzopertha dominica* and *Tribolium castaneum* showed 100% mortality under 24 h EF treatment, while *S. oryzae* survived in the immature stages [37]. In another study of *S. oryzae* and *T. castaneum*, the tolerance of eggs was the lowest and that of the pupae was the highest [22]. In this study, the PH_3_-sensitive *S. oryzae* and PH_3_-resistant *S. oryzae* were treated with PH_3_, EF, and MB fumigants, and the PH_3_-resistant *S. oryzae* was treated with EF. The lowest tolerance was confirmed in eggs.

There is a growing worldwide problem of PH_3_ resistant grain pests, and PH_3_ resistant *S. oryzae* has also been reported in various countries. As a result of the FAO test (0.04 g/m^3^) on *S. oryzae*, *R. dominica*, and *T. castaneum* for the detection of PH_3_ resistance in Morocco, 18 of 19 groups of *S. oryzae* showed PH_3_ resistance [18]. As PH_3_-resistant *S. oryzae* is increasing, FAO test was performed to confirm the PH_3_ resistant *S. oryzae* of South Korea. Here, we report the first confirmed the existence of PH_3_-resistant *S. oryzae* in South Korea. In general, exposure time of pests to PH_3_ takes three to five days to have an insecticidal effect, while exposure time of *Sitophilus* sp. to PH_3_ requires 12 days or more [13,38]. Chaudhry [13] found that resistant pests did not meet sufficient resistance levels at past disinfection sites, even with the long-term use of phosphine to control *S. oryzae*, but under insufficient disinfection time, some surviving insects have rebuilt their population. Insecticides must be applied repeatedly at overdose levels to cause resistance. Therefore, the airtightness of fumigation chamber should be maintained and fumigation time should be considered when applying PH_3_ in the field.

Efficacy of PH_3_ was found to be affected by the exposure time more than the concentration [39]. Therefore, in this study, the concentration was fixed and the treatment time was varied to control PH_3_-resistant *S. oryzae*. PH_3_-resistant *S. oryzae* treated with 0.5 mg/L PH_3_ for 502 h was able to achieve 100% mortality. Therefore, PH_3_ should be exposed for approximately 21 days to control PH_3_-resistant *S. oryzae*. PH_3_ could be used to treat the PH_3_ resistant *S. oryzae* for 100% mortality rate, but a long exposure time is needed. It cannot be used in the field because of long exposure time. Therefore EF, which shows effect in short time, can used to control PH_3_ resistant *S. oryzae* instead of PH_3_. EF was first reported to be effective in box-car grain fumigation in Australia, and the use of EF in grains has been proposed for the last 40 years, but it has been difficult to apply in the field because of the disadvantages of low insecticidal activity and high flammability compared to other fumigants [40]. In this study, EF was treated at 20 °C, and 90 mg/L or more had to be added to control all of the PH_3_-susceptible and -resistant *S. oryzae*. Since EF has a low explosion threshold (85 g/t), more studies should be conducted to increase the efficacy of EF at lower concentration to avoid explosion.

## 5. Conclusions

EF was used as a substitute to treat PH_3_-susceptible and -resistant *S. oryzae*, but EF required a higher concentration of fumigant than that of other fumigants. To use EF to control *S. oryzae*, it is necessary to carry out studies to increase the insecticidal effect at a low concentration.

## Figures and Tables

**Table 1:** Lethal concentration time (LCT) of *S. oryzae* exposed to EF fumigant at 20 °C in 12 L desiccator.

Strain ^a^	Stages	*n*	LCT_50_ (mg h/L) (95% CL ^b^)	RR_50_^c^	LCT_99_ (mg h/L) (95% CL)	RR_99_	Slope ± SE	*df*	χ^2^
S	Egg	542	75.795 (35.294–99.852)	1.00	186.623 (162.962–233.473)	1.00	5.946 ± 0.921	5	0.00
	Early larva	450	60.110 (40.398–78.077)	1.00	213.792 (160.004–346.215)	1.00	4.222 ± 0.332	4	0.04
	Late larva	600	160.491 (97.945–265.422)	1.00	775.478 (389.069–14622.164)	1.00	3.401 ± 0.789	6	8.13
	Pupa	600	255.797 (209.902–340.130)	1.00	2373.015 (1289.504–6737.892)	1.00	2.405 ± 0.233	6	0.80
	Adult	540	77.711 (58.090–100.392)	1.00	316.190 (204.109–837.332)	1.00	3.818 ± 0.494	5	1.97
R	Egg	760	60.034 (2.415–98.658)	0.79	217.692 (127.285–2673.091)	1.17	4.159 ± 1.378	7	40.65
	Early larva	630	64.450 (45.514–81.298)	1.07	311.913 (224.910–557.809)	1.46	3.398 ± 0.396	6	5.58
	Late larva	540	149.028 (85.417–191.420)	0.93	449.200 (289.551–5031.511)	0.58	4.856 ± 1.089	5	17.00
	Pupa	540	140.408 (53.470–187.556)	0.55	312.447 (217.735–10214.042)	0.13	6.698 ± 1.307	5	7.38
	Adult	540	66.043 (43.299–87.325)	0.85	394.584 (266.764–807.344)	1.25	2.997 ± 0.32	5	3.27

^a^ Strain = S; Susceptible, R; Resistance. ^b^ CL = confidence limits. ^c^ RR = LCT of the R strain/LCT of the S strain.

**Table 2:** Lethal concentration time (LCT) of *S. oryzae* exposed to MB fumigant at 20 °C in 12 L desiccator.

Strain ^a^	Stages	*n*	LCT_50_ (mg h/L) (95% CL ^b^)	RR_50_^c^	LCT_99_ (mg h/L) (95% CL)	RR_99_	Slope ± SE	*df*	χ^2^
S	Egg	576	9.997 (7.125–12.255)	1.00	24.111 (21.131–28.229)	1.00	6.086 ± 0.554	5	0.041
	Early larva	540	12.113 (7.018–14.187)	1.00	19.929 (17.331–30.090)	1.00	10.762 ± 2.137	5	10.61
	Late larva	540	18.952 (15.578–21.901)	1.00	60.351 (49.342–82.503)	1.00	4.626 ± 0.373	5	0.53
	Pupa	540	21.104 (11.907–29.393)	1.00	67.795 (44.055–255.928)	1.00	4.591 ± 0.826	5	30.65
	Adult	540	17.824 (16.772–18.433)	1.00	22.297 (20.853–26.931)	1.00	23.929 ± 3.761	5	0.04
R	Egg	545	17.842 (6.726–22.183)	1.78	24.683 (16.463–27.220)	1.02	16.509 ± 3.323	5	0.00
	Early larva	540	14.900 (13.461–17.159)	1.23	34.098 (26.534–53.390)	1.71	6.471 ± 0.601	5	0.96
	Late larva	630	25.840 (21.031–32.636)	1.36	68.905 (48.205–161.691)	1.14	5.462 ± 0.863	6	17.13
	Pupa	450	43.520 (35.203–73.578)	2.06	73.072 (50.790–186.850)	1.08	10.339 ± 1.080	4	0.43
	Adult	540	16.397 (14.842–17.388)	0.92	23.792 (21.992–27.719)	1.07	14.392 ± 1.655	5	0.00

^a^ Strain = S; Susceptible, R; Resistance. ^b^ CL = confidence limits. ^c^ RR = LCT of the R strain/LCT of the S strain.

**Table 3:** Lethal concentration time (LCT) of *S. oryzae* exposed to PH_3_ fumigant at 20 °C in 12 L desiccator.

Strain ^a^	Stages	*n*	LCT_50_ (mg h/L) (95% CL ^b^)	RR_50_^c^	LCT_99_ (mg h/L) (95% CL)	RR_99_	Slope ± SE	*df*	χ^2^
S	Egg	540	0.440 (0.042–0.861)	1.00	4.625 (2.911–17.448)	1.00	2.277 ± 0.425	5	7.73
	Early larva	540	0.602 (0.307–0.907)	1.00	12.243 (7.436–29.192)	1.00	1.779 ± 0.173	5	0.55
	Late larva	540	3.901 (2.799–5.862)	1.00	119.032 (49.421–529.324)	1.00	1.567 ± 0.133	5	2.33
	Pupa	632	6.171 (3.462–11.297)	1.00	2450.358 (536.688–9983.186)	1.00	0.895 ± 0.099	6	6.90
	Adult	540	0.295 (0.225–0.342)	1.00	0.634 (0.555–0.797)	1.00	6.996 ± 0.776	5	1.37
R	Egg	717	6.595 (0.773–13.142)	14.99	57.206 (42.018–99.369)	12.37	2.480 ± 0.512	7	1.03
	Early larva	725	28.456 (0.943–45.574)	47.27	107.914 (60.927–276614.079)	8.81	4.019 ± 1.390	7	67.32
	Late larva	633	48.170 (26.561–105.326)	12.35	241.311 (108.523–5887.895)	2.03	3.325 ± 0.845	6	25.57
	Pupa	630	29.106 (24.576–33.114)	4.72	91.760 (77.518–116.869)	0.04	4.666 ± 0.364	6	2.71
	Adult	540	16.550 (12.113–19.290)	56.10	25.938 (22.889–30.448)	40.91	11.924 ± 1.581	5	0.26

^a^ Strain = S; Susceptible, R; Resistance. ^b^ CL = confidence limits. ^c^ RR = LCT of the R strain/LCT of the S strain.
